# Curative effect of immediate reconstruction after neoadjuvant chemotherapy for breast cancer: a systematic review and meta-analysis

**DOI:** 10.3389/fonc.2023.1288744

**Published:** 2023-11-23

**Authors:** Gang Li, Hongxiang Ji, Jiang Li, Linfeng Xiao, Zhan Chen

**Affiliations:** Department of General Surgery, The Chenggong Hospital Affiliated to Xiamen University, Xiamen, China

**Keywords:** breast cancer, immediate reconstruction, mastectomy, neoadjuvant chemotherapy, meta-analysis

## Abstract

**Background:**

The safety of mastectomy (MT) with immediate reconstruction (IR) in breast cancer patients who have completed neoadjuvant chemotherapy (NAC) is not apparent. This meta-analysis aims to systematically evaluate the differences in surgical complications and postoperative survival rates between MT with IR (MT+IR) and MT alone in post-NAC breast cancer patients.

**Methods:**

The PubMed, Embase, Cochrane Library, WanFang Data, and CNKI databases were systematically searched, and cohort studies of post-NAC breast cancer patients with MT+IR or MT surgery were collected from databases inception to May 25, 2023. Two researchers independently executed literature screening, data extraction, and bias risk assessment, and meta-analysis was performed using Revman 5.3 software.

**Results:**

A total of 12 studies involving 7378 cases who have accepted NAC were collected for this study. The results showed that compared with the MT group, the relative risk of surgical complications in the MT+IR group was increased by 44%, with no statistical significant [RR=1.44, 95% CI (0.99, 2.09), P=0.06]. While among study subgroups with a median follow-up of less than one year, more surgical complications occurred in the MT+IR group by 23% [RR=1.23, 95% CI (1.00, 1.52), P=0.05]. There was no significant differences in overall survival, disease-free survival, local relapse-free survival, and distant metastasis-free survival between the two groups.

**Conclusions:**

Compared with the MT, MT+IR does not affect the postoperative survival rate in post-NAC breast cancer patients, accompanied by a mild increase in short-term surgical complications, but no significant difference in long-term complications.

**Systematic review registration:**

https://www.crd.york.ac.uk/prospero, identifier CRD42023421150.

## 1 Introduction

Breast cancer (BC) is the most popular carcinoma among females worldwide (1). Most of these patients need a mastectomy (MT). Whereas patients who experienced MT, which often requires the removal of the entire breast, may experience long-term negative impacts on their physical and mental health, and their treatment compliance may be reduced (2, 3). MT with immediate reconstruction (MT+IR) has been shown to significantly improve a patient’s quality of life by recent researches (4–6). Therefore, MT+IR has become a popular alternative to maintain the breast’s appearance and improve patients’ quality of life (7).

Neoadjuvant chemotherapy (NAC) is a critical element of systematic breast cancer treatment and is associated with improved survival compared to adjuvant chemotherapy in some breast cancer patients (8, 9). In early breast cancer, NAC can make breast-conserving surgery (BCS) more feasible than the same chemotherapy given after surgery (10, 11). The increasing use of NAC has led to a rapid worldwide increase in the rate of BCS over the past few decades (12–15). However, there is a certain proportion of patients still not suitable for BCS (12, 13, 16, 17). Some patients eligible for BCS post-NAC still chose MT and MT plus reconstructive surgery (18–21). In such cases, MT+IR presents an attractive alternative to BCS as it can help avoid psychosocial morbidity and suboptimal cosmetic outcomes (5, 22). In recent years, the proportion of reconstruction has increased yearly, accompanied by the incidence of complications decreasing (23–25). Due to the lack of high-quality evidence, the safety of IR in post-NAC is still controversial. In Japan, there is a considerable disparity in doctors’ opinion of the safety of IR, with nearly one-quarter of doctors believing that IR could adversely impact patient prognosis (26).

Currently, there are no available RCT researches on the effect of MT+IR following NAC. Previous studies have primarily focused on comparing the outcomes of MT+IR after NAC and adjuvant therapy after MT+IR (27–29). However, these studies do not provide sufficient information for breast cancer patients who have completed NAC and are preparing for operation.

It is necessary to conduct a meta-analysis of the differences in surgical complications and postoperative survival between MT+IR and MT alone after NAC. We aimed to provide more reference data for breast cancer patients who are not candidates for BCS after NAC.

## 2 Methods

This meta-analysis was conducted according to the Preferred Reporting Items for Systematic Reviews and Meta-analyses (PRISMA) standards (30), and the protocol was registered in the PROSPERO database (CRD42023421150).

### 2.1 Literature search

Two independent researchers searched PubMed, Embase, the Cochrane Library, the Wanfang database, and the CNKI database for studies on breast cancer patients who underwent MT combined with or without IR surgery after NAC. The retrieval time limit was from the establishment of the database to May 25, 2023. The index words used were as follows: “Mammaplasty”, “Breast Implantation” and “Neoadjuvant Therapy”. An approach involving the combination of subject words and free words was adopted in the retrieval (**Supplementary Table 1**).

### 2.2 Inclusion and exclusion criteria

The inclusion criteria were as follows: (1) cohort studies or randomized control studies; (2) patients with breast cancer who underwent breast surgery after NAC; (3) comparison of the MT+IR with the MT; and (4) report of relevant outcomes, including overall survival (OS), disease-free survival (DFS), local recurrence-free survival (LRFS), distant metastasis-free survival (DMFS), and surgical complications.

Studies were excluded if they met the following criteria: (1) literature in languages other than Chinese and English; (2) no outcome indicators mentioned above; (3) repeat studies; (4) uncontrol studies; (5) study without valid data or data that could not be extracted; and (6) abstracts, lectures, conference abstracts, and incomplete data.

### 2.3 Risk of bias assessment

The included studies used the Newcastle Ottawa Scale (NOS) to assess the risk of bias (31). Two independent researchers conducted the cross-check. If the NOS score was ≥6, the study’s quality was considered high.

### 2.4 Data extraction

Two independent researchers extracted the data, such as the general information, specific intervention measures, number of cases in the MT+IR and MT groups, total number, publication time, research time, first author, and number of complications. The comparison of survival data (OS, DFS, LRFS, and DMFS) between the two groups used the hazard ratio (HR). If the HR value and 95% CI were directly reported in the literature or the survival rates of the two groups at multiple time points were reported, the ln(HR) and SE[ln(HR)] of the OS, DFS, LRFS, and DMFS between the two groups could be calculated by using the Excel attachment calculations spreadsheet provided by Tierney et al. (32) If the survival curves of OS, DFS, LRFS, and DMFS of the two groups were reported in the literature, the survival data were extracted using Engauge Digitizer version 4.1 software and then calculated using the Excel attachment calculations spreadsheet provided by Tierney et al. (32) We finally used the ln(HR) and SE[ln(HR)] from each study for meta-analysis.

### 2.5 Statistical analysis

Data were analyzed by RevMan5.3 software. The relative risk (RR) was used as the effective index for count data, and HR was used as the effective index for survival data. The heterogeneity between the results of the studies was assessed using χ2 inspection analysis, with the inspection level set at α=0.10, combined with I2 to determine the heterogeneity size. The fixed effect model was used when the homogeneity of the results was not significant (I^2^<50%, P≥0.05). The random effect model was used when the heterogeneity test showed that the heterogeneity of the results was statistically significant (I^2^≥50%, P<0.05), and the source of heterogeneity was further analyzed. Sensitivity analysis was used to evaluate the stability of the results using the one-by-one exclusion method.

## 3 Results

### 3.1 Literature screening

**Figure 1:**
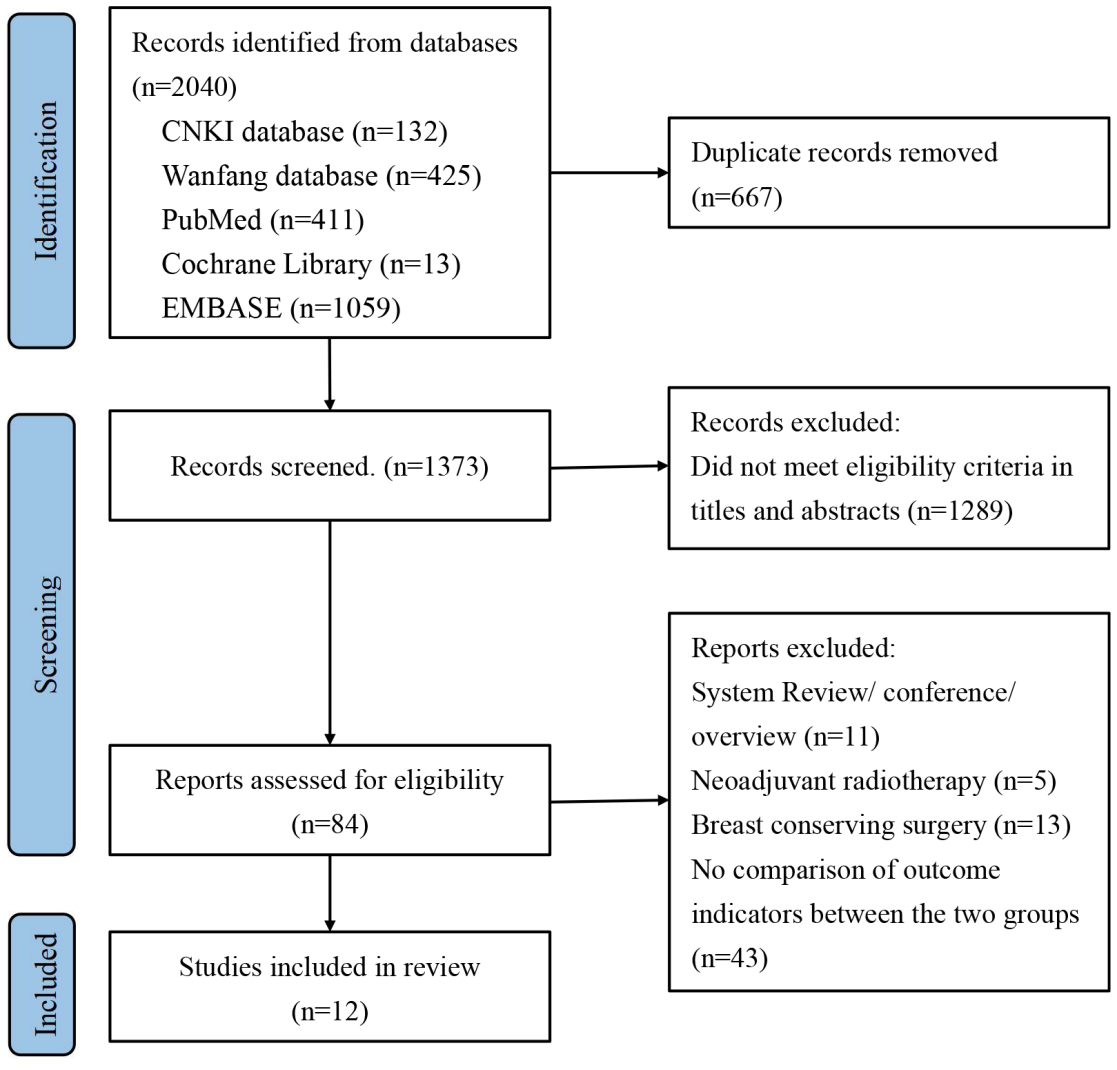
Preferred Reporting Items for Systematic Review and Meta-Analysis of Diagnostic Test Accuracy Studies (PRISMA) flow diagram for study selection.

Initially, we identified a total of 2040 articles from various databases, including 132 articles from the CNKI database, 425 articles from the Wanfang database, 411 articles from the PubMed database, 13 articles from the Cochrane Library, and 1059 articles from the Embase database. After screening and reviewing the title, abstract and full text, we included 12 cohort studies involving 7378 patients (33–44). The literature screening process is shown in **Figure 1**.

### 3.2 Study characteristics and risk of bias

The 12 included studies comprised 2019 patients with MT+IR and 5359 patients with MT. Bias risk assessment showed that the NOS scores of all 12 studies were ≥6, and the studies were regarded as high-quality research (**Table 1**).

**Table 1:** Basic characteristics of the included studies.

First author, year	Study type	Study time	Country	MT+IR/MT(n)	Match the propensity scores	Operation program of MT+IR group	Outcomes	Median follow-up	NOS score
Gouy 2005 (33)	R	1985-1995	France	48/181	no	MT	LRFS/DMFS	10 years	6
Golshan 2011 (34)	P	2004-2008	USA	13/24	no	MT	Complication	1 year	7
Prabhu 2012 (35)	R	1997-2010	USA	40/60	no	SSM	Complication	31.6 months/30 months	8
Kansal 2013 (36)	P	2007-2010	USA	62/57	yes	MT	Complication	1 year	7
Abt 2014 (37)	R	2005-2011	USA	820/2876	no	MT	Complication	30 days	6
Aurilio 2014 (38)	R	1995-2006	Italy, Europe	59/74	no	MT	OS/DFS	8.2 years	9
Gerber 2014 (39)	R	2007-2010	Germany, Switzerl	54/142	no	MT	Complication	12 weeks	6
Ryu 2017 (40)	R	2008-2015	Korea	31/85	yes	NSM/SSM	OS/DFS/DMFS/LRFS	29.2 months/38.8 months	9
Vieira 2019 (41)	R	2005-2011	Brazil	48/96	yes	NSM/SSM	OS/DFS/LRFS	75.9 months/67 months	8
Wu 2020 (42)	R	2010-2016	Korea	323/323	yes	NSM/SSM	OS/DFS/DMFS/LRFS	67 months/68 months	9
Park 2021 (43)	R	2008-2014	Korea	345/1354	no	MT	OS	30.1 months	8
Wu 2022 (44)	R	2010-2016	Korea	209/209	yes	NSM	OS/DFS/DMFS/LRFS	70 months/74months	9

R, Retrospective cohort study; P, Prospective cohort study; MT, mastectomy; IR, immediate reconstruction; NSM, nipple-sparing mastectomy; SSM, skin sparing mastectomy; OS, overall survival; DFS, disease free survival; LRFS, local recurrence free survival; DMFS, distant metastasis free survival; NOS, Newcastle−Ottawa scale.

### 3.3 Surgical complications

#### 3.3.1 Meta-analysis results

**Figure 2:**
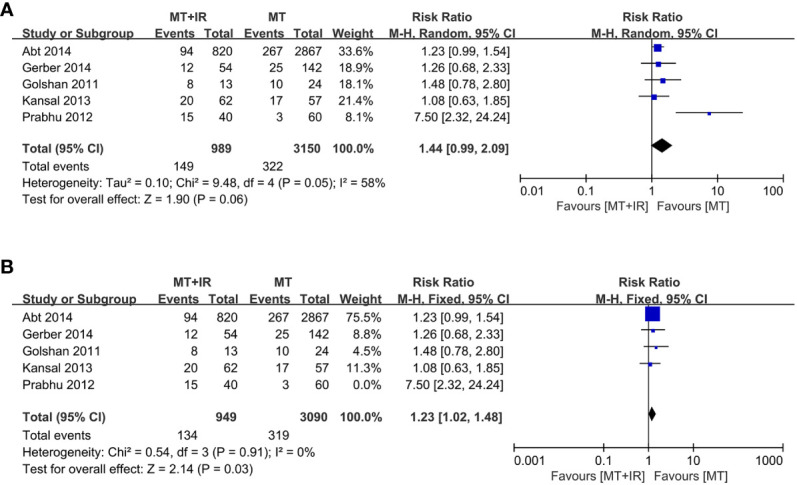
Forest plot of surgical complication in two groups. **(A)** All of five studies was included. **(B)** The study of Prabhu et al. was excluded. MT, Mastectomy; IR, Immediate reconstruction.

A total of five studies reported surgical complications between the two groups (34–37, 39), including 989 patients in the MT+IR group and 3150 patients in the MT group. Meta-analysis using the random effect model showed no significant difference in the incidence of complications between the two groups [RR=1.44, 95% CI (0.99, 2.09), P=0.06] (**Figure 2A**).

#### 3.3.2 Subgroup analysis of surgical complications

**Figure 3:**
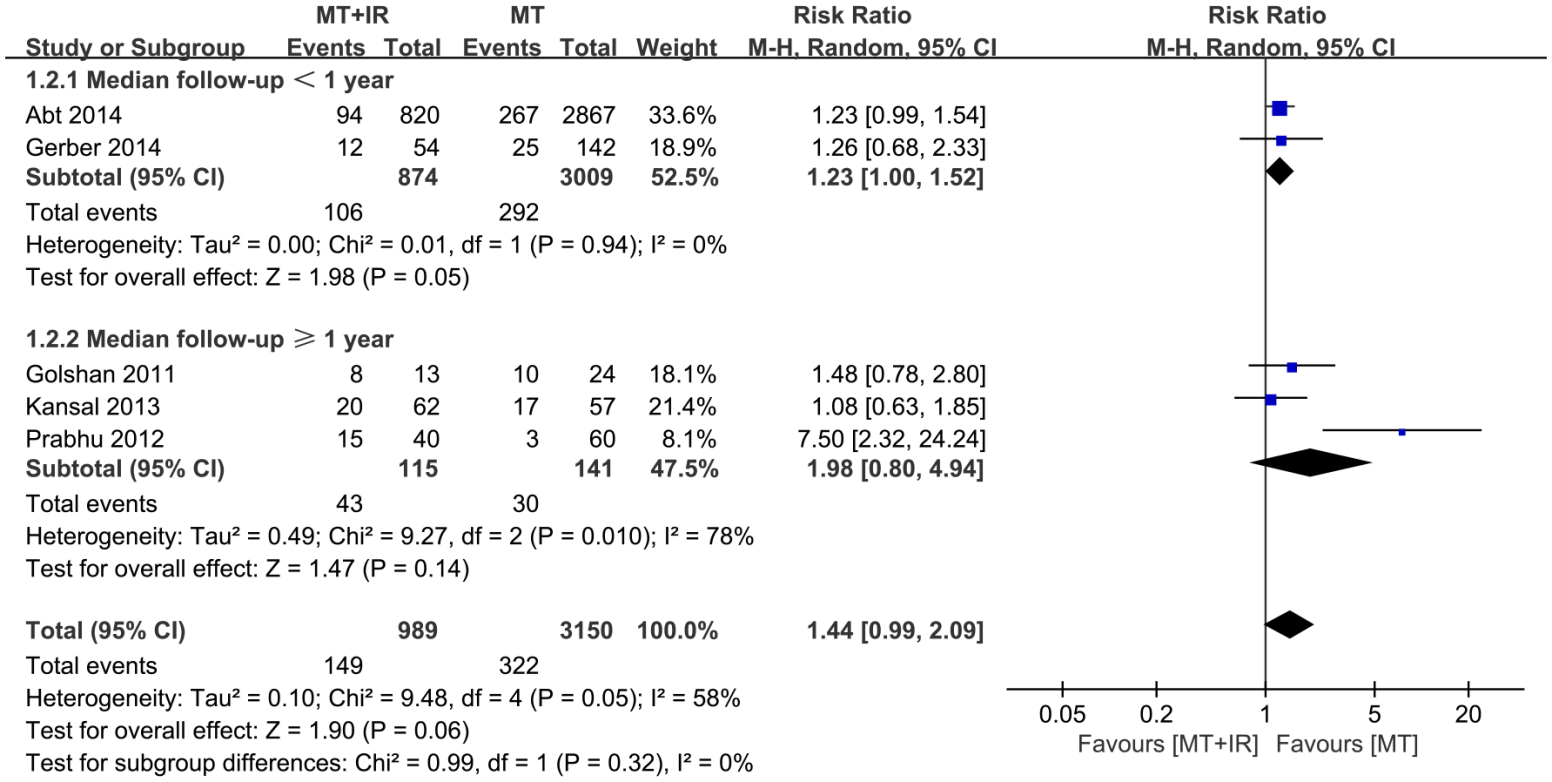
Subgroup analysis of different follow-up time on surgical complications.

According to different median follow-up times, subgroup analysis was conducted on surgical complications. Among study subgroups with a median follow-up of less than one year, more surgical complications occurred in the MT+IR group [RR=1.23, 95% CI (1.00, 1.52), P=0.05]. However, in the study subgroup with a longer median follow-up, there was no significant difference in the incidence of surgical complications between the two groups [RR=1.98, 95% CI (0.80, 4.94), P=0.14] (**Figure 3**).

Subgroup analysis of surgical complications in the MT+IR and IR groups was performed according to whether the propensity score matched. There were no significant differences in surgical complications, regardless of propensity score matching (**Supplementary Figure 1**).

### 3.4 Survival

#### 3.4.1 OS

**Figure 4:**
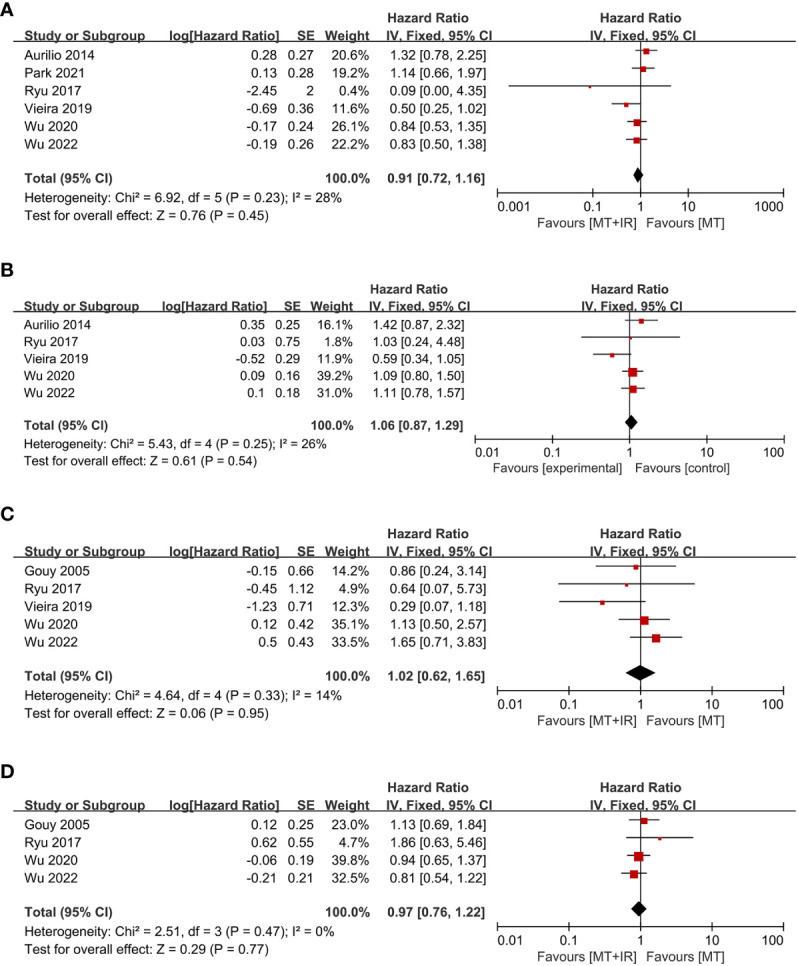
Forest plot of postoperative survival condition in two groups. **(A)** Overall survival; **(B)** Disease-free survival; **(C)** Local recurrence-free survival; **(D)** Distant metastasis-free survival. MT, Mastectomy; IR, Immediate reconstruction.

Six studies compared postoperative OS between the two groups (38, 40–44), including 982 MT+IR patients and 2028 MT patients. Meta-analysis using a fixed effect model showed no significant difference in the OS between the two groups [HR=0.91, 95% CI (0.72, 1.16), P=0.45] (**Figure 4A**).

#### 3.4.2 DFS

Five studies compared postoperative DFS between the two groups (38, 40–42, 44), including 670 MT+IR patients and 787 MT patients. Meta-analysis using a fixed effect model showed no significant difference in the DFS between the two groups [HR=1.06, 95% CI (0.87, 1.29), P=0.54] (**Figure 4B**).

#### 3.4.3 LRFS

Five studies compared postoperative LRFS between the two groups (33, 40–42, 44), including 659 MT+IR patients and 894 MT patients. Meta-analysis using a fixed effect model showed no significant difference in the LRFS between the two groups [HR=1.02, 95% CI (0.62, 1.65), P=0.95] (**Figure 4C**).

#### 3.4.4 DMFS

Four studies compared postoperative DMFS between the two groups (33, 40, 42, 44), including 611 MT+IR patients and 798 MT patients. Meta-analysis using a fixed effect model showed no significant difference in the DMFS between the two groups [HR=0.97, 95% CI (0.76, 1.22), P=0.77] (**Figure 4D**).

#### 3.4.5 Subgroup analysis of survival

Subgroup analysis of OS, DFS, LRFS, and DMFS in the MT+IR and IR groups was performed according to whether the propensity score matched. Among each subgroup, there were no significant differences in OS, DFS, LRFS, and DMFS between the two groups (**Supplementary Figures 2-5**).

### 3.5 Sensitivity analysis

We conducted a sensitivity analysis by excluding one study at a time. When the survey of Prabhu et al. (35) was excluded, the statistical heterogeneity of the meta-analysis results decreased significantly (I^2^ = 0%, P=0.91). The results significantly differed in the incidence of surgical complications between the MT+IR group and the MT group [RR=1.23, 95% CI (1.02, 1.48), P=0.03] (**Figure 2B**).

## 4 Discussion

Breast reconstruction has been widely accepted as a mean to enhance breast cancer patients’ quality of life, mental well-being, and aesthetics degree post-surgery as evidenced by recent studies (45, 46). Our study provides valuable information that MT+IR in breast cancer patients after NAC may bring more short-term surgical complications than MT. The results of previous studies have been controversial on whether IR increases surgical complications. Mortenson et al. (47) and Lee et al. (48) both observed an increased incidence of wound complications when IR was combined with MT. The study of Hamahata et al. (49) yielded reports of a 10.0% postoperative complication rate in the IR group versus 6.1% in the non-IR group. A network meta-analysis of 51 studies revealed that the risk of overall complications and surgical site infection was more significant in the MT+IR group than in the MT group (50). Conversely, other studies found no significant difference in the incidence of complications between the two groups (51, 52).

A meta-analysis investigated the incidence of complications between MT+IR after NAC and adjuvant chemotherapy after MT+IR. There was no significant difference in the incidence of complications between the two groups (27). However, when the implant reconstruction subgroup was analyzed, there was some evidence suggesting that implant losses were more likely to occur in patients post-NAC compared to those in control groups (27). Another meta-analysis included 26 studies comparing surgical complications in breast cancer patients with or without NAC who underwent any breast surgery (29). In that study, it was found that NAC did not increase the risk of certain complications, including seroma, wound complications, skin or nipple necrosis, flap ischemia or loss, and implant loss (29). However, these studies have limited reference significance for patients who have completed or underwent NAC when formulating the following surgery scheme. Matar et al. (53) showed that compared with MT+IR, delayed reconstruction (DR) after MT has a lower incidence of surgical complications, especially hematoma and postoperative infection. Although there is no significant correlation between the occurrence of surgical complications and the recurrence rate and mortality of breast cancer, DR may be a better alternative for patients afraid of complications (54). Among the five studies that investigated complications we included, only Abt et al. (37) studied wound and systemic complications containing Accordion Expanded grades 1 to 6 but did not report detailed numbers of occurrence of each complication (55, 56). The other four studies examined only wound complications that included complications of Accordion Expanded grades 1 to 4 (34–36, 39).

Without considering the influence of NAC, several previous meta-analyses proved that there was no significant difference in postoperative DFS, OS, and local recurrence rate between the MT+IR group and the MT group (57, 58). However, Shen et al. (50) conducted a Bayesian analysis and concluded that the OS of the MT+IR group was more advantageous than that of the MT group. Generally, there is a biased selection in the MT+IR group, for the patients may be younger or have higher schooling, some of them have a lower clinical stage and a better response to NAC (41, 59). Baseline characteristics of patients before NAC and the response to NAC can affect the prognosis of patients (60, 61). Based on this selective factor, some studies showed that the MT+IR group had higher OS and LRFS and a lower recurrence rate (62, 63). However, when propensity score matching was used, the survival rate between the two groups did not show significant differences in many studies. Lee et al. (64, 65) compared DMFS and breast cancer-specific survival rates between the two groups after propensity score matching, and the results showed no significant difference. Yi et al. (66) found no significant difference in the DFS between the two groups after adjusting for the clinical TNM staging. A study by Song et al. (67) showed that in patients with tumor sizes greater than 3 cm, the DFS of the MT group was higher than the MT+IR group, especially in HER2-positive and triple-negative patients. We performed subgroup analyses of propensity-matched studies that were matched for age, clinical stage, molecular type, and response to NAC (40–42, 44). Similar to other studies, our study showed no significant difference between the two groups in OS, DFS, LRFS and DMFS, regardless of propensity score matching (**Supplementary Figures 2-5**).

There is no suitable report to provide a reference for the conclusion of the effect of IR on prognosis in post-NAC patients. Although no difference in prognosis was observed in our study, the accuracy is also limited by the bias caused by retrospective analysis. Liu et al. (28) demonstrated the same OS benefits for both NAC and non-NAC cases in patients with breast cancer receiving MT+IR. However, some studies suggest that MT+IR patients who received NAC had worse OS than MT+IR patients without NAC (68). It is necessary to consider the patient’s response to NAC, as patients with pathologic complete response after NAC have a better prognosis than patients with limited or no response (41, 69, 70). Only a few studies have matched this factor, which could decrease the influence of different factors. After matching patients in the MT+IR and MT groups based on their responsiveness to NAC, Vieira et al. (41) found no statistically significant difference in DFS and LRFS between the two groups. However, the MT+IR group had a better OS and cancer-related survival, which they still attributed to selecting patients with a better response to NAC for IR. Ryu et al. (40) proved that OS, DFS, DMFS, and LRFS did not differ significantly between the two groups, whose matched variables included age, clinical stage before NAC, response to NAC, and pathologic stage after NAC. Two studies from Korea matched the response to NAC and also found no significant differences in OS, DFS, DMFS, and LRFS between the two groups, even in patients with locally advanced breast cancer (42, 44). In addition, some studies have shown that the best operation time after NAC is 4-8 weeks because it is related to increased OS and DFS and reduced complications (71, 72). However, due to the lack of relevant data, this study did not further analyze subgroups.

The heterogeneity test results comparing surgical complications between the two groups revealed significant heterogeneity among the studies. When the study of Prabhu et al. (35) was excluded, the heterogeneity decreased significantly, suggesting that this study may be one of the sources of heterogeneity. Further data analysis indicated that the patients had locally advanced breast cancer, and the surgical method in the MT+IR group was skin-sparing mastectomy (SSM). In contrast, other studies employed nipple-sparing mastectomy (NSM), SSM, and traditional MT as surgical methods in the MT+IR group. SSM/NSM retains a portion of the native breast structure, resulting in better breast appearance and quality of life for the patients. However, it may also bring about more surgical complication (73). Future research needs to analyze the specific surgical scheme after differentiation.

This study has the following limitations: (1) the investigation was conducted with a limited number of studies, which may present a risk of publication bias; (2) most of the included studies were retrospective studies, which may have selection bias and retrospective bias; (3) the long-term cosmetic effects of the two groups were not studied; (4) because the radiotherapy data in each study could not be extracted, our study did not consider radiotherapy, which may introduce bias; and (5) it is necessary to organize criteria related to complications of breast surgery as the number of patients submitted to IR is increasing, and the complications are decreasing yearly.

It is impossible to perform prospective randomized studies related to oncoplastic surgery because we can not randomize the type of breast surgery, and matched studies represent the best study form. It is necessary to take more studies matched by the response to NAC and other baseline characteristics with adequate follow-up to evaluate the long-term results of MT+IR after NAC. Further standardization of surgical complications and IR categories must be studied to obtain the most suitable and safe reconstruction method for breast cancer patients after NAC. The long-term cosmetic and symmetrization rates of IR in post-NAC patients need further evaluation.

## 5 Conclusion

Our meta-analysis demonstrated that compared with the MT, MT+IR does not affect the postoperative OS, DFS, LRFS, and DMFS in post-NAC breast cancer patients, accompanied by a mild increase in short-term surgical complications, but no significant difference in long-term complications.

## Data availability statement

The original contributions presented in the study are included in the article/**Supplementary Material**. Further inquiries can be directed to the corresponding author.

## Author contributions

GL: Conceptualization, Data curation, Formal Analysis, Investigation, Methodology, Project administration, Software, Validation, Visualization, Writing – original draft, Writing – review and editing, Funding acquisition, Resources. HJ: Writing – original draft, Writing – review and editing, Conceptualization, Data curation, Funding acquisition, Investigation, Methodology, Resources, Software, Validation, Visualization. JL: Conceptualization, Writing – review and editing, Investigation, Supervision. LX: Writing – review and editing, Conceptualization, Investigation, Supervision. ZC: Writing – review and editing, Conceptualization, Data curation, Formal Analysis, Funding acquisition, Investigation, Methodology, Project administration, Resources, Validation.

## References

[1] Feng K Jia Z Liu G Xing Z Li J Li J . A review of studies on omitting surgery after neoadjuvant chemotherapy in breast cancer. Am J Cancer Res (2022) 12(8):3512–31. PMC9442028 36119847

[2] McKevitt E Saleeb M Liu G Warburton R Pao J Dingee C . Differences in preoperative health-related quality of life between women receiving mastectomy or breast conserving surgery in a prospectively recruited cohort of breast cancer patients. Curr Oncol (2022) 30(1):118–29. doi: 10.3390/curroncol30010010 PMC9857337 36661659

[3] Fortin J Leblanc M Elgbeili G Cordova M Marin M Brunet A . The mental health impacts of receiving a breast cancer diagnosis: A meta-analysis. Br J Cancer (2021) 125(11):1582–92. doi: 10.1038/s41416-021-01542-3 PMC8608836 34482373

[4] Rautalin M Jahkola T Roine R . Breast reconstruction-prospective follow up on breast cancer patients' Health-related quality of life. World J Surg (2022) 46(4):836–44. doi: 10.1007/s00268-021-06426-4 PMC8885544 35001140

[5] Clarijs ME Peeters N van Dongen SAF Koppert LB Pusic AL Mureau MAM . Quality of life and complications after nipple- versus skin-sparing mastectomy followed by immediate breast reconstruction: A systematic review and meta-analysis. Plast Reconstr Surg (2023) 152(1):12e–24e. doi: 10.1097/prs.0000000000010155 PMC10298179 36728484

[6] De La Cruz L Moody AM Tappy EE Blankenship SA Hecht EM . Overall survival, disease-free survival, local recurrence, and nipple-areolar recurrence in the setting of nipple-sparing mastectomy: A meta-analysis and systematic review. Ann Surg Oncol (2015) 22(10):3241–9. doi: 10.1245/s10434-015-4739-1 26242363

[7] Zhang C Jiang H . Effect of immediate breast reconstruction after standardized breast cancer surgery on the quality of life of patients: A prospective multicenter study. J Healthc Eng (2021) 2021:2840043. doi: 10.1155/2021/2840043 34737848 PMC8563118

[8] Fisher CS Ma CX Gillanders WE Aft RL Eberlein TJ Gao F . Neoadjuvant chemotherapy is associated with improved survival compared with adjuvant chemotherapy in patients with triple-negative breast cancer only after complete pathologic response. Ann Surg Oncol (2012) 19(1):253–8. doi: 10.1245/s10434-011-1877-y PMC3892697 21725686

[9] Zhao Y Chen L Zheng X Shi Y . Quality of life in patients with breast cancer with neoadjuvant chemotherapy: A systematic review. BMJ Open (2022) 12(11):1–9. doi: 10.1136/bmjopen-2022-061967 PMC9677026 36400735

[10] Brown L Carr M Sam C Sun W Whiting J Kim Y . Tolerance and outcomes of neoadjuvant chemotherapy in geriatric breast cancer patients. J Surg Res (2023) 283:329–35. doi: 10.1016/j.jss.2022.10.092 36427442

[11] (EBCTCG) EBCTCG . Long-term outcomes for neoadjuvant versus adjuvant chemotherapy in early breast cancer: meta-analysis of individual patient data from ten randomised trials. Lancet Oncol (2018) 19(1):27–39. doi: 10.1016/s1470-2045(17)30777-5 29242041 PMC5757427

[12] Petruolo O Sevilimedu V Montagna G Le T Morrow M Barrio AV . How often does modern neoadjuvant chemotherapy downstage patients to breast-conserving surgery? Ann Surg Oncol (2021) 28(1):287–94. doi: 10.1245/s10434-020-08593-5 PMC7722183 32514804

[13] Pfob A Dubsky P . The underused potential of breast conserving therapy after neoadjuvant system treatment - causes and solutions. Breast (2023) 67:110–5. doi: 10.1016/j.breast.2023.01.008 PMC9982288 36669994

[14] Lautner M Lin H Shen Y Parker C Kuerer H Shaitelman S . Disparities in the use of breast-conserving therapy among patients with early-stage breast cancer. JAMA Surg (2015) 150(8):778–86. doi: 10.1001/jamasurg.2015.1102 PMC4712635 26083835

[15] Zhang BL Sivasubramaniam PG Zhang Q Wang J Zhang B Gao JD . Trends in radical surgical treatment methods for breast Malignancies in China: A multicenter 10-year retrospective study. Oncologist (2015) 20(9):1036–43. doi: 10.1634/theoncologist.2014-0281 PMC4571796 26253559

[16] Criscitiello C Golshan M Barry WT Viale G Wong S Santangelo M . Impact of neoadjuvant chemotherapy and pathological complete response on eligibility for breast-conserving surgery in patients with early breast cancer: A meta-analysis. Eur J Cancer (2018) 97:1–6. doi: 10.1016/j.ejca.2018.03.023 29734046

[17] Li Y Chen H He J Fan Z Zhang H . The outcome of neoadjuvant chemotherapy and the current trend of surgical treatment in young women with breast cancer: A multicenter real-world study (Csbrs-012). Front Public Health (2023) 11:1100421. doi: 10.3389/fpubh.2023.1100421 36895689 PMC9988895

[18] Kummerow KL Du L Penson DF Shyr Y Hooks MA . Nationwide trends in mastectomy for early-stage breast cancer. JAMA Surg (2015) 150(1):9–16. doi: 10.1001/jamasurg.2014.2895 25408966

[19] Chang YK Co M Kwong A . Conversion rate from mastectomy to breast conservation after neoadjuvant dual target therapy for her2-positive breast cancer in the Asian population. Breast Cancer (2020) 27(3):456–63. doi: 10.1007/s12282-019-01037-3 31916189

[20] Yamaguchi T Hozumi Y Sagara Y Takahashi M Yoneyama K Fujisawa T . The impact of neoadjuvant systemic therapy on breast conservation rates in patients with her2-positive breast cancer: surgical results from a phase ii randomized controlled trial. Surg Oncol (2021) 36:51–5. doi: 10.1016/j.suronc.2020.11.008 33310293

[21] Golshan M Cirrincione CT Sikov WM Carey LA Berry DA Overmoyer B . Impact of neoadjuvant therapy on eligibility for and frequency of breast conservation in stage ii-iii her2-positive breast cancer: surgical results of calgb 40601 (Alliance). Breast Cancer Res Treat (2016) 160(2):297–304. doi: 10.1007/s10549-016-4006-6 27704226 PMC5189982

[22] Heneghan HM Prichard RS Lyons R Regan PJ Kelly JL Malone C . Quality of life after immediate breast reconstruction and skin-sparing mastectomy - a comparison with patients undergoing breast conserving surgery. Eur J Surg Oncol (2011) 37(11):937–43. doi: 10.1016/j.ejso.2011.08.126 21899982

[23] O'Halloran N Lowery A Kalinina O Sweeney K Malone C McLoughlin R . Trends in breast reconstruction practices in a specialized breast tertiary referral centre. BJS Open (2017) 1(5):148–57. doi: 10.1002/bjs5.23 PMC5989961 29951617

[24] Young WA Degnim AC Hoskin TL Jakub JW Nguyen MD Tran NV . Outcomes of > 1300 nipple-sparing mastectomies with immediate reconstruction: the impact of expanding indications on complications. Ann Surg Oncol (2019) 26(10):3115–23. doi: 10.1245/s10434-019-07560-z 31342370

[25] Jonczyk MM Jean J Graham R Chatterjee A . Surgical Trends in Breast Cancer: A Rise in Novel Operative Treatment Options over a 12 year Analysis. Breast Cancer Res Treat (2019) 173(2):267–74. doi: 10.1007/s10549-018-5018-1 PMC6486837 30361873

[26] Yamakado R Ishitobi M Kondo N Yamauchi C Sasada S Nogi H . Physicians' Perception about the impact of breast reconstruction on patient prognosis: A survey in Japan. Breast Cancer (2023) 30(2):302–8. doi: 10.1007/s12282-022-01421-6 PMC9758461 36527601

[27] Varghese J Gohari S Rizki H Faheem M Langridge B Kümmel S . A systematic review and meta-analysis on the effect of neoadjuvant chemotherapy on complications following immediate breast reconstruction. Breast (2021) 55:55–62. doi: 10.1016/j.breast.2020.11.023 33341706 PMC7750646

[28] Liu C Yang J Tsai I Hsu C Yean L Hung C . Comparison of Overall Survival after Neoadjuvant and Adjuvant Chemotherapy in Patients with Early Breast Cancer with Immediate Breast Reconstruction after Mastectomy: A Retrospective, Matched Case-Control Study. Oncol Lett (2022) 24(6):437. doi: 10.3892/ol.2022.13557 36420073 PMC9641822

[29] Lorentzen T Heidemann LN Möller S Bille C . Impact of neoadjuvant chemotherapy on surgical complications in breast cancer: A systematic review and meta-analysis. Eur J Surg Oncol (2022) 48(1):44–52. doi: 10.1016/j.ejso.2021.09.007 34548216

[30] Page MJ McKenzie JE Bossuyt PM Boutron I Hoffmann TC Mulrow CD . The prisma 2020 statement: an updated guideline for reporting systematic reviews. Bmj (2021) 372:n71. doi: 10.1136/bmj.n71 33782057 PMC8005924

[31] Stang A . Critical evaluation of the newcastle-ottawa scale for the assessment of the quality of nonrandomized studies in meta-analyses. Eur J Epidemiol (2010) 25(9):603–5. doi: 10.1007/s10654-010-9491-z 20652370

[32] Tierney J Stewart L Ghersi D Burdett S Sydes M . Practical methods for incorporating summary time-to-event data into meta-analysis. Trials (2007) 8:16. doi: 10.1186/1745-6215-8-16 17555582 PMC1920534

[33] Gouy S Rouzier R Missana MC Atallah D Youssef O Barreau-Pouhaer L . Immediate reconstruction after neoadjuvant chemotherapy: effect on adjuvant treatment starting and survival. Ann Surg Oncol (2005) 12(2):161–6. doi: 10.1245/aso.2005.04.003 15827797

[34] Golshan M Garber JE Gelman R Tung N Smith BL Troyan S . Does neoadjuvant bevacizumab increase surgical complications in breast surgery? Ann Surg Oncol (2011) 18(3):733–7. doi: 10.1245/s10434-010-1366-8 20882415

[35] Prabhu R Godette K Carlson G Losken A Gabram S Fasola C . The impact of skin-sparing mastectomy with immediate reconstruction in patients with stage iii breast cancer treated with neoadjuvant chemotherapy and postmastectomy radiation. Int J Radiat Oncol Biol Phys (2012) 82(4):e587–93. doi: 10.1016/j.ijrobp.2011.09.024 22197232

[36] Kansal KJ Dominici LS Tolaney SM Isakoff SJ Smith BL Jiang W . Neoadjuvant bevacizumab: surgical complications of mastectomy with and without reconstruction. Breast Cancer Res Treat (2013) 141(2):255–9. doi: 10.1007/s10549-013-2682-z 24026859

[37] Abt NB Flores JM Baltodano PA Sarhane KA Abreu FM Cooney CM . Neoadjuvant chemotherapy and short-term morbidity in patients undergoing mastectomy with and without breast reconstruction. JAMA Surg (2014) 149(10):1068–76. doi: 10.1001/jamasurg.2014.1076 PMC4352300 25133469

[38] Aurilio G Bagnardi V Graffeo R Nolè F Petit J Locatelli M . Does immediate breast reconstruction after mastectomy and neoadjuvant chemotherapy influence the outcome of patients with non-endocrine responsive breast cancer? Anticancer Res (2014) 34(11):6677–83. 25368274

[39] Gerber B von Minckwitz G Eidtmann H Rezai M Fasching P Tesch H . Surgical outcome after neoadjuvant chemotherapy and bevacizumab: results from the geparquinto study (Gbg 44). Ann Surg Oncol (2014) 21(8):2517–24. doi: 10.1245/s10434-014-3606-9 24740826

[40] Ryu JM Park S Paik HJ Nam SJ Kim SW Lee SK . Oncologic safety of immediate breast reconstruction in breast cancer patients who underwent neoadjuvant chemotherapy: short-term outcomes of a matched case-control study. Clin Breast Cancer (2017) 17(3):204–10. doi: 10.1016/j.clbc.2016.10.009 28065399

[41] Vieira R Ribeiro L Carrara G Abrahão-MaChado L Kerr L Nazário A . Effectiveness and safety of implant-based breast reconstruction in locally advanced breast carcinoma: A matched case-control study. Breast Care (Basel) (2019) 14(4):200–10. doi: 10.1159/000496429 PMC6751465 31558894

[42] Wu ZY Kim HJ Lee JW Chung IY Kim JS Lee SB . Long-term oncologic outcomes of immediate breast reconstruction vs conventional mastectomy alone for breast cancer in the setting of neoadjuvant chemotherapy. JAMA Surg (2020) 155(12):1142–50. doi: 10.1001/jamasurg.2020.4132 PMC7557719 33052412

[43] Park S Jeong J Han W Lee Y Kim H Lee S . Is mastectomy with immediate reconstruction safe for patients undergoing neoadjuvant chemotherapy? A nationwide study from Korean breast cancer society. Breast Cancer (2021) 28(4):874–83. doi: 10.1007/s12282-021-01223-2 33586091

[44] Wu ZY Han HH Kim HJ Chung IY Kim J Lee SB . A propensity score-matched analysis of long-term oncologic outcomes after nipple-sparing versus conventional mastectomy for locally advanced breast cancer. Ann Surg (2022) 276(2):386–90. doi: 10.1097/sla.0000000000004416 33201107

[45] Schmidt J Wetzel C Lange K Heine N Ortmann O . Patients' Experience of breast reconstruction after mastectomy and its influence on postoperative satisfaction. Arch Gynecol Obstet (2017) 296(4):827–34. doi: 10.1007/s00404-017-4495-5 28864887

[46] Kim MK Kim T Moon HG Jin US Kim K Kim J . Effect of cosmetic outcome on quality of life after breast cancer surgery. Eur J Surg Oncol (2015) 41(3):426–32. doi: 10.1016/j.ejso.2014.12.002 25578249

[47] Mortenson MM Schneider PD Khatri VP Stevenson TR Whetzel TP Sommerhaug EJ . Immediate breast reconstruction after mastectomy increases wound complications: however, initiation of adjuvant chemotherapy is not delayed. Arch Surg (2004) 139(9):988–91. doi: 10.1001/archsurg.139.9.988 15381618

[48] Lee J Lee SK Kim S Koo MY Choi MY Bae SY . Does immediate breast reconstruction after mastectomy affect the initiation of adjuvant chemotherapy? J Breast Cancer (2011) 14(4):322–7. doi: 10.4048/jbc.2011.14.4.322 PMC3268930 22323920

[49] Hamahata A Kubo K Takei H Saitou T Hayashi Y Matsumoto H . Impact of immediate breast reconstruction on postoperative adjuvant chemotherapy: A single center study. Breast Cancer (2015) 22(3):287–91. doi: 10.1007/s12282-013-0480-4 23756827

[50] Shen Z Sun J Yu Y Chiu C Zhang Z Zhang Y . Oncological safety and complication risks of mastectomy with or without breast reconstruction: A bayesian analysis. J Plast Reconstr Aesthet Surg (2021) 74(2):290–9. doi: 10.1016/j.bjps.2020.08.121 33093010

[51] Zhong T Hofer SO McCready DR Jacks LM Cook FE Baxter N . A comparison of surgical complications between immediate breast reconstruction and mastectomy: the impact on delivery of chemotherapy–an analysis of 391 procedures. Ann Surg Oncol (2012) 19(2):560–6. doi: 10.1245/s10434-011-1950-6 21792509

[52] Eriksen C Frisell J Wickman M Lidbrink E Krawiec K Sandelin K . Immediate reconstruction with implants in women with invasive breast cancer does not affect oncological safety in a matched cohort study. Breast Cancer Res Treat (2011) 127(2):439–46. doi: 10.1007/s10549-011-1437-y 21409394

[53] Matar DY Wu M Haug V Orgill DP Panayi AC . Surgical complications in immediate and delayed breast reconstruction: A systematic review and meta-analysis. J Plast Reconstr Aesthet Surg (2022) 75(11):4085–95. doi: 10.1016/j.bjps.2022.08.029 36202732

[54] Cui W Xie Y . Oncological results in women with wound complications following mastectomy and immediate breast reconstruction: A meta-analysis. Int Wound J (2023) 20(5):1361–8. doi: 10.1111/iwj.13982 PMC10088858 36336978

[55] Strasberg SM Linehan DC Hawkins WG . The accordion severity grading system of surgical complications. Ann Surg (2009) 250(2):177–86. doi: 10.1097/SLA.0b013e3181afde41 19638919

[56] Jończyk J Jankau J . Accordion: A useful and workable classification of complications after breast reconstructive surgery. Plast Surg (Oakv) (2022) 30(3):197–203. doi: 10.1177/22925503211008439 35990398 PMC9389063

[57] Yang X Zhu C Gu Y . The prognosis of breast cancer patients after mastectomy and immediate breast reconstruction: A meta-analysis. PloS One (2015) 10(5):e0125655. doi: 10.1371/journal.pone.0125655 26024490 PMC4449019

[58] Zhang P Li CZ Wu CT Jiao GM Yan F Zhu HC . Comparison of immediate breast reconstruction after mastectomy and mastectomy alone for breast cancer: A meta-analysis. Eur J Surg Oncol (2017) 43(2):285–93. doi: 10.1016/j.ejso.2016.07.006 27503441

[59] Roder D Zorbas H Kollias J Pyke C Walters D Campbell I . Factors predictive of immediate breast reconstruction following mastectomy for invasive breast cancer in Australia. Breast (2013) 22(6):1220–5. doi: 10.1016/j.breast.2013.09.011 24128741

[60] Harbeck N . Neoadjuvant and adjuvant treatment of patients with her2-positive early breast cancer. Breast (2022) 62 Suppl 1(Suppl 1):S12–s6. doi: 10.1016/j.breast.2022.01.006 PMC9097807 35148934

[61] Heil J Kuerer HM Pfob A Rauch G Sinn HP Golatta M . Eliminating the breast cancer surgery paradigm after neoadjuvant systemic therapy: current evidence and future challenges. Ann Oncol (2020) 31(1):61–71. doi: 10.1016/j.annonc.2019.10.012 31912797

[62] Carter SA Lyons GR Kuerer HM Bassett RL Jr. Oates S Thompson A . Operative and oncologic outcomes in 9861 patients with operable breast cancer: single-institution analysis of breast conservation with oncoplastic reconstruction. Ann Surg Oncol (2016) 23(10):3190–8. doi: 10.1245/s10434-016-5407-9 27406093

[63] Lanitis S Tekkis PP Sgourakis G Dimopoulos N Al Mufti R Hadjiminas DJ . Comparison of skin-sparing mastectomy versus non-skin-sparing mastectomy for breast cancer: A meta-analysis of observational studies. Ann Surg (2010) 251(4):632–9. doi: 10.1097/SLA.0b013e3181d35bf8 20224371

[64] Lee S Lee J Kim H Ko B Son B Eom J . Long-term outcomes of patients with breast cancer after nipple-sparing mastectomy/skin-sparing mastectomy followed by immediate transverse rectus abdominis musculocutaneous flap reconstruction: comparison with conventional mastectomy in a single center study. Medicine (2018) 97(18):e0680. doi: 10.1097/md.0000000000010680 29718895 PMC6393080

[65] Lee S Lee J Son B Eom J Kim E Lee T . Oncologic safety of skin-sparing mastectomy followed by immediate reconstruction in young patients with breast cancer. Asian J Surg (2019) 42(1):274–82. doi: 10.1016/j.asjsur.2018.04.004 29908898

[66] Yi M Kronowitz S Meric-Bernstam F Feig B Symmans W Lucci A . Local, regional, and systemic recurrence rates in patients undergoing skin-sparing mastectomy compared with conventional mastectomy. Cancer (2011) 117(5):916–24. doi: 10.1002/cncr.25505 PMC4371507 20945319

[67] Song Y Sun S Li D Han J Niu M Luo S . Long-term oncologic safety of immediate reconstructive surgery in patients with invasive breast cancer: A retrospective matched-cohort study. World J Surg Oncol (2021) 19(1):348. doi: 10.1186/s12957-021-02450-9 34930333 PMC8686330

[68] Grigor EJM Stein MJ Arnaout A Ghaedi B Ramsay T Zhang J . The effect of neoadjuvant chemotherapy on safety outcomes following immediate breast reconstruction. J Plast Reconstr Aesthet Surg (2022) 75(8):2520–5. doi: 10.1016/j.bjps.2022.02.048 35396192

[69] Kong X Moran MS Zhang N Haffty B Yang Q . Meta-analysis confirms achieving pathological complete response after neoadjuvant chemotherapy predicts favourable prognosis for breast cancer patients. Eur J Cancer (2011) 47(14):2084–90. doi: 10.1016/j.ejca.2011.06.014 21737257

[70] Li X Dai D Chen B Tang H Wei W . Oncological outcome of complete response after neoadjuvant chemotherapy for breast conserving surgery: A systematic review and meta-analysis. World J Surg Oncol (2017) 15(1):210. doi: 10.1186/s12957-017-1273-6 29183336 PMC5706340

[71] Cullinane C Shrestha A Al Maksoud A Rothwell J Evoy D Geraghty J . Optimal timing of surgery following breast cancer neoadjuvant chemotherapy: A systematic review and meta-analysis. Eur J Surg Oncol (2021) 47(7):1507–13. doi: 10.1016/j.ejso.2021.01.025 33589241

[72] Thawanyarat K Johnstone T Rowley M Navarro Y Hinson C Nazerali RS . Optimizing postoperative outcomes following neoadjuvant chemotherapy and mastectomy with immediate reconstruction: A national analysis. J Surg Oncol (2023) 127(5):768–75. doi: 10.1002/jso.27196 36602535

[73] Agha RA Al Omran Y Wellstead G Sagoo H Barai I Rajmohan S . Systematic review of therapeutic nipple-sparing versus skin-sparing mastectomy. BJS Open (2019) 3(2):135–45. doi: 10.1002/bjs5.50119 PMC6433323 30957059

