# Supplementary material for: Curative effect of immediate reconstruction after neoadjuvant chemotherapy for breast cancer: a systematic review and meta-analysis
Source: Front Oncol. 2023 Nov 23;13:1288744. doi: 10.3389/fonc.2023.1288744 (PMC10702342; doi:10.3389/fonc.2023.1288744)
Supplement: Supplementary file 1 [file DataSheet_1.docx]

Supplementary Material

# Supplementary Data

## Supplementary Table 1 Search strategy for each database

| Databases | Search Strategy |
| --- | --- |
| CNKI Database | TKA='乳腺癌' AND TKA='新辅助' AND TKA='手术' AND( TKA='重建' OR TKA='移植' OR TKA='假体') |
| WanFang Database | 摘要:(新辅助) and (摘要:(重建) or 摘要:(假体) or 摘要:(移植)) and (摘要:(乳房) or 摘要:(乳腺癌)) |
| PubMed | ("Mammaplasty"[MeSH Terms] OR "Breast Implantation"[MeSH Terms] OR "Mammaplasties"[Title/Abstract] OR "Mammoplasty"[Title/Abstract] OR "Mammoplasties"[Title/Abstract] OR "breast reconstruction"[Title/Abstract] OR "breast reconstructions"[Title/Abstract] OR "reconstruction breast"[Title/Abstract] OR "reconstructions breast"[Title/Abstract]) **AND** ( "Neoadjuvant Therapy"[MeSH Terms] OR "neoadjuvant"[Title/Abstract] OR "Neoadjuvant Therapies"[Title/Abstract] OR "Therapy, Neoadjuvant"[Title/Abstract] OR "Neoadjuvant Treatment"[Title/Abstract] OR "Neoadjuvant Treatments"[Title/Abstract] OR "Treatment, Neoadjuvant"[Title/Abstract] OR "Neoadjuvant Chemoradiotherapy"[Title/Abstract] OR "Chemoradiotherapy, Neoadjuvant"[Title/Abstract] OR "Neoadjuvant Chemoradiotherapies"[Title/Abstract] OR "Neoadjuvant Chemoradiation Therapy"[Title/Abstract] OR "Chemoradiation Therapy, Neoadjuvant"[Title/Abstract] OR "Neoadjuvant Chemoradiation Therapies"[Title/Abstract] OR "Therapy, Neoadjuvant Chemoradiation"[Title/Abstract] OR "Neoadjuvant Chemoradiation Treatment"[Title/Abstract] OR "Chemoradiation Treatment, Neoadjuvant"[Title/Abstract] OR "Neoadjuvant Chemoradiation Treatments"[Title/Abstract] OR "Treatment, Neoadjuvant Chemoradiation"[Title/Abstract] OR "Neoadjuvant Chemoradiation"[Title/Abstract] OR "Chemoradiation, Neoadjuvant"[Title/Abstract] OR "Neoadjuvant Chemoradiations"[Title/Abstract] OR "Neoadjuvant Radiotherapy"[Title/Abstract] OR "Neoadjuvant Radiotherapies"[Title/Abstract] OR "Radiotherapy, Neoadjuvant"[Title/Abstract] OR "Neoadjuvant Radiation Treatment"[Title/Abstract] OR "Neoadjuvant Radiation Treatments"[Title/Abstract] OR "Radiation Treatment, Neoadjuvant"[Title/Abstract] OR "Treatment, Neoadjuvant Radiation"[Title/Abstract] OR "Neoadjuvant Radiation Therapy"[Title/Abstract] OR "Neoadjuvant Radiation Therapies"[Title/Abstract] OR "Radiation Therapy, Neoadjuvant"[Title/Abstract] OR "Therapy, Neoadjuvant Radiation"[Title/Abstract] OR "Neoadjuvant Radiation"[Title/Abstract] OR "Neoadjuvant Radiations"[Title/Abstract] OR "Radiation, Neoadjuvant"[Title/Abstract] OR "Neoadjuvant Chemotherapy"[Title/Abstract] OR "Chemotherapy, Neoadjuvant"[Title/Abstract] OR "Neoadjuvant Chemotherapies"[Title/Abstract] OR "Neoadjuvant Chemotherapy Treatment"[Title/Abstract] OR "Chemotherapy Treatment, Neoadjuvant"[Title/Abstract] OR "Neoadjuvant Chemotherapy Treatments"[Title/Abstract] OR "Treatment, Neoadjuvant Chemotherapy"[Title/Abstract] OR "Neoadjuvant Systemic Therapy"[Title/Abstract] OR "Neoadjuvant Systemic herapies"[Title/Abstract] OR "Systemic Therapy, Neoadjuvant"[Title/Abstract] OR "Therapy, Neoadjuvant Systemic"[Title/Abstract] OR "Neoadjuvant Systemic Treatment"[Title/Abstract] OR "Neoadjuvant Systemic Treatments"[Title/Abstract] OR "Systemic Treatment, Neoadjuvant"[Title/Abstract] OR "Treatment, Neoadjuvant Systemic"[Title/Abstract]) |
| Embase | #1 'breast reconstruction'/exp OR 'mammaplasty'/exp OR 'breast implantation'/exp  #2 mammaplasty:ab,ti OR mammaplasties:ab,ti OR mammoplasty:ab,ti OR mammoplasties:ab,ti OR 'breast reconstruction':ab,ti OR 'breast reconstructions':ab,ti OR 'reconstruction, breast':ab,ti OR 'reconstructions, breast':ab,ti  #3 #1 OR #2  #4 neoadjuvant:ab,ti OR 'neoadjuvant therapy'/exp  #5 'Neoadjuvant Therapies':ab,ti OR 'Therapy, Neoadjuvant':ab,ti OR 'Neoadjuvant Treatment':ab,ti OR 'Neoadjuvant Treatments':ab,ti OR 'Treatment, Neoadjuvant':ab,ti OR 'Neoadjuvant Chemoradiotherapy':ab,ti OR 'Chemoradiotherapy, Neoadjuvant':ab,ti OR 'Neoadjuvant Chemoradiotherapies':ab,ti OR 'Neoadjuvant Chemoradiation Therapy':ab,ti OR 'Chemoradiation Therapy, Neoadjuvant':ab,ti OR 'Neoadjuvant Chemoradiation Therapies':ab,ti OR 'Therapy, Neoadjuvant Chemoradiation':ab,ti OR 'Neoadjuvant Chemoradiation Treatment':ab,ti OR 'Chemoradiation Treatment, Neoadjuvant':ab,ti OR 'Neoadjuvant Chemoradiation Treatments':ab,ti OR 'Treatment, Neoadjuvant Chemoradiation':ab,ti OR 'Neoadjuvant Chemoradiation':ab,ti OR 'Chemoradiation, Neoadjuvant':ab,ti OR 'Neoadjuvant Chemoradiations':ab,ti OR 'Neoadjuvant Radiotherapy':ab,ti OR 'Neoadjuvant Radiotherapies':ab,ti OR 'Radiotherapy, Neoadjuvant':ab,ti OR 'Neoadjuvant Radiation Treatment':ab,ti OR 'Neoadjuvant Radiation Treatments':ab,ti OR 'Radiation Treatment, Neoadjuvant':ab,ti OR 'Treatment, Neoadjuvant Radiation':ab,ti OR 'Neoadjuvant Radiation Therapy':ab,ti OR 'Neoadjuvant Radiation Therapies':ab,ti OR 'Radiation Therapy, Neoadjuvant':ab,ti OR 'Therapy, Neoadjuvant Radiation':ab,ti OR 'Neoadjuvant Radiation':ab,ti OR 'Neoadjuvant Radiations':ab,ti OR 'Radiation, Neoadjuvant':ab,ti OR 'Neoadjuvant Chemotherapy':ab,ti OR 'Chemotherapy, Neoadjuvant':ab,ti OR 'Neoadjuvant Chemotherapies':ab,ti OR 'Neoadjuvant Chemotherapy Treatment':ab,ti OR 'Chemotherapy Treatment, Neoadjuvant':ab,ti OR 'Neoadjuvant Chemotherapy Treatments':ab,ti OR 'Treatment, Neoadjuvant Chemotherapy':ab,ti OR 'Neoadjuvant Systemic Therapy':ab,ti OR 'Neoadjuvant Systemic herapies':ab,ti OR 'Systemic Therapy, Neoadjuvant':ab,ti OR 'Therapy, Neoadjuvant Systemic':ab,ti OR 'Neoadjuvant Systemic Treatment':ab,ti OR 'Neoadjuvant Systemic Treatments':ab,ti OR 'Systemic Treatment, Neoadjuvant':ab,ti OR 'Treatment, Neoadjuvant Systemic':ab,ti  #6 #4 OR #5  #7 #3 AND #6 |
| The Cochrane Library | #1 MeSH descriptor:[Mammaplasty] explode all trees MeSH  #2 MeSH descriptor:[Neoadjuvant Therapy] explode all trees MeSH  #3 #1 AND #2 Limits |

## Supplementary Table 2 Total evaluation scores of Newcastle-Ottawa Scale of each study regarding selection, comparability and outcome ascertainment

| Number | First author/year | Selection | Comparability | Outcome ascertainment | Bias risk (Total scores) |
| --- | --- | --- | --- | --- | --- |
| 1 | Gouy 2005 | 4 | 1 | 1 | 6 |
| 2 | Golshan 2011 | 4 | 0 | 3 | 7 |
| 3 | Prabhu 2012 | 4 | 1 | 3 | 8 |
| 4 | Kansal 2013 | 4 | 0 | 3 | 7 |
| 5 | Abt 2014 | 4 | 0 | 2 | 6 |
| 6 | Aurilio 2014 | 4 | 2 | 3 | 9 |
| 7 | Gerber 2014 | 4 | 0 | 2 | 6 |
| 8 | Ryu 2017 | 4 | 2 | 3 | 9 |
| 9 | Vieira 2019 | 4 | 1 | 3 | 8 |
| 10 | Wu 2020 | 4 | 2 | 3 | 9 |
| 11 | Park 2021 | 4 | 1 | 3 | 8 |
| 12 | Wu 2022 | 4 | 2 | 3 | 9 |

# Supplementary Figures

#
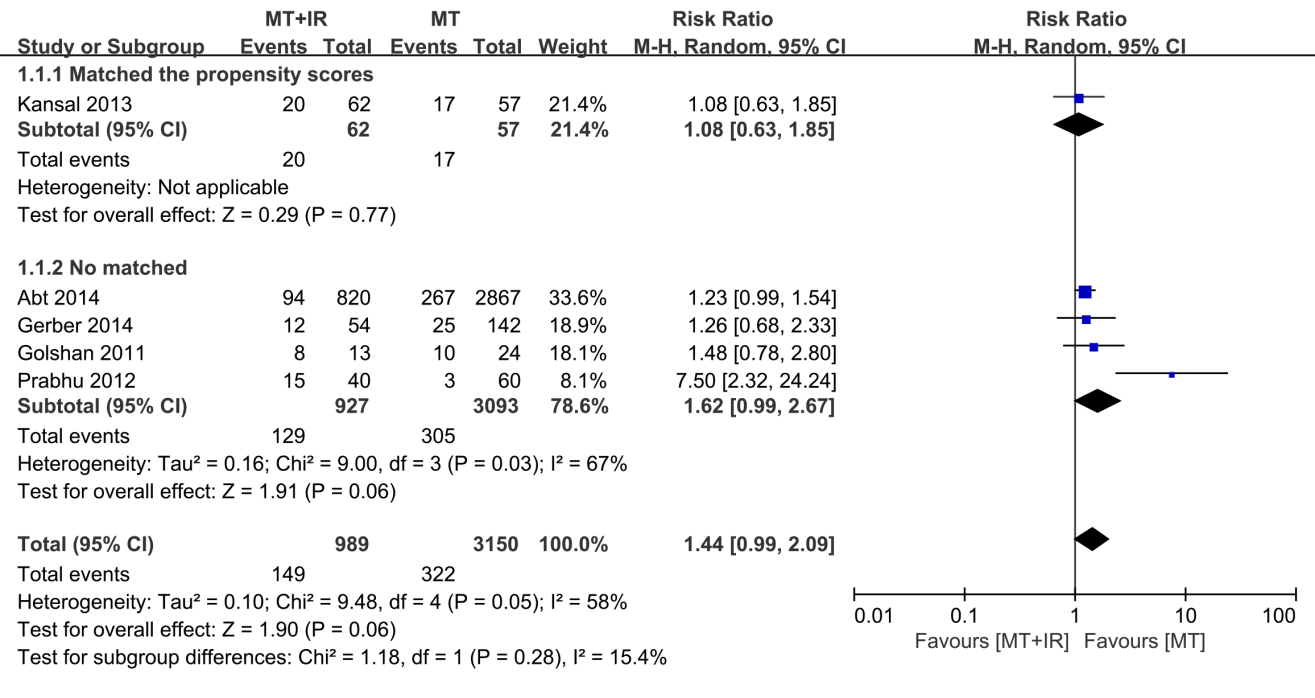


# **Supplementary Figure 1.** Subgroup analysis of complication according to whether or not the propensity score was matched.


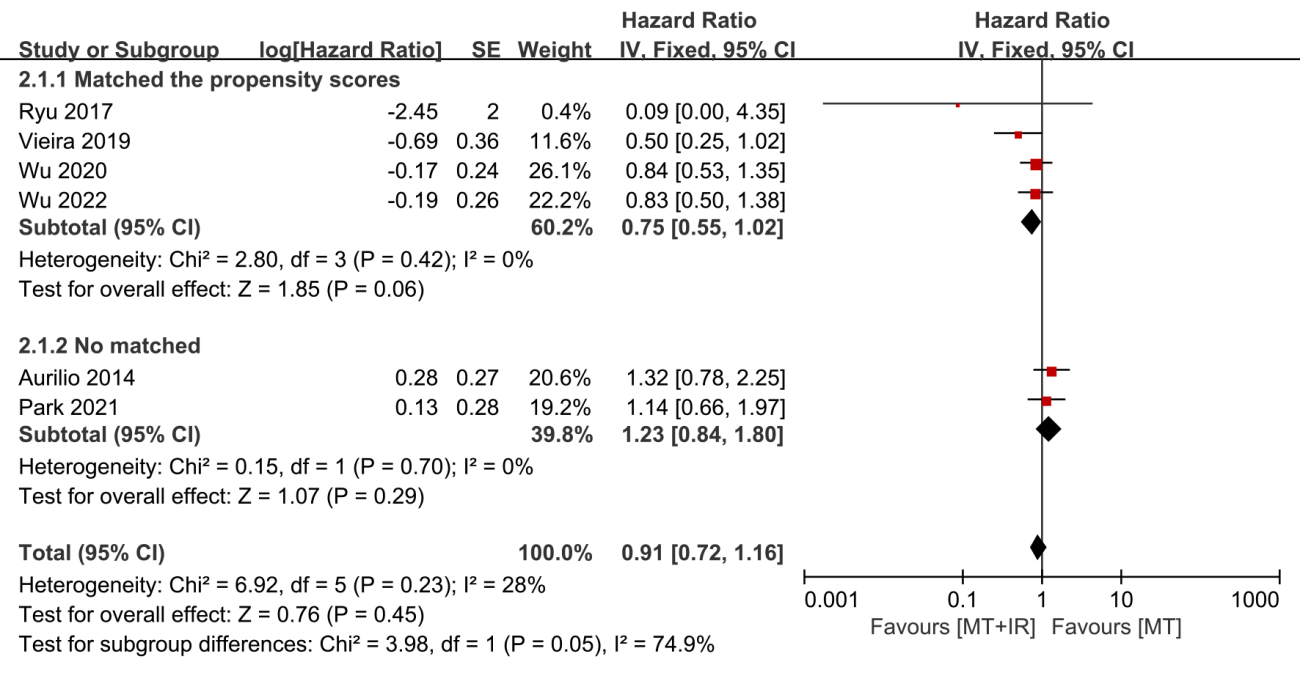


**Supplementary Figure 2.** Subgroup analysis of OS according to whether or not the propensity score was matched.


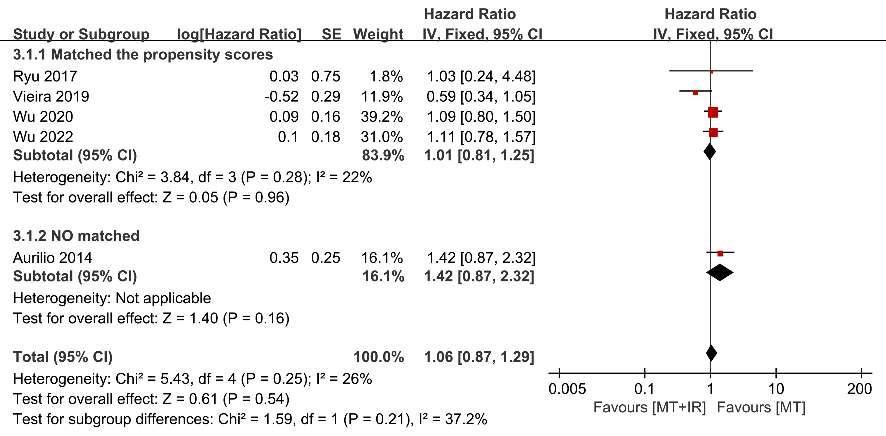


**Supplementary Figure 3.** Subgroup analysis of DFS according to whether or not the propensity score was matched.


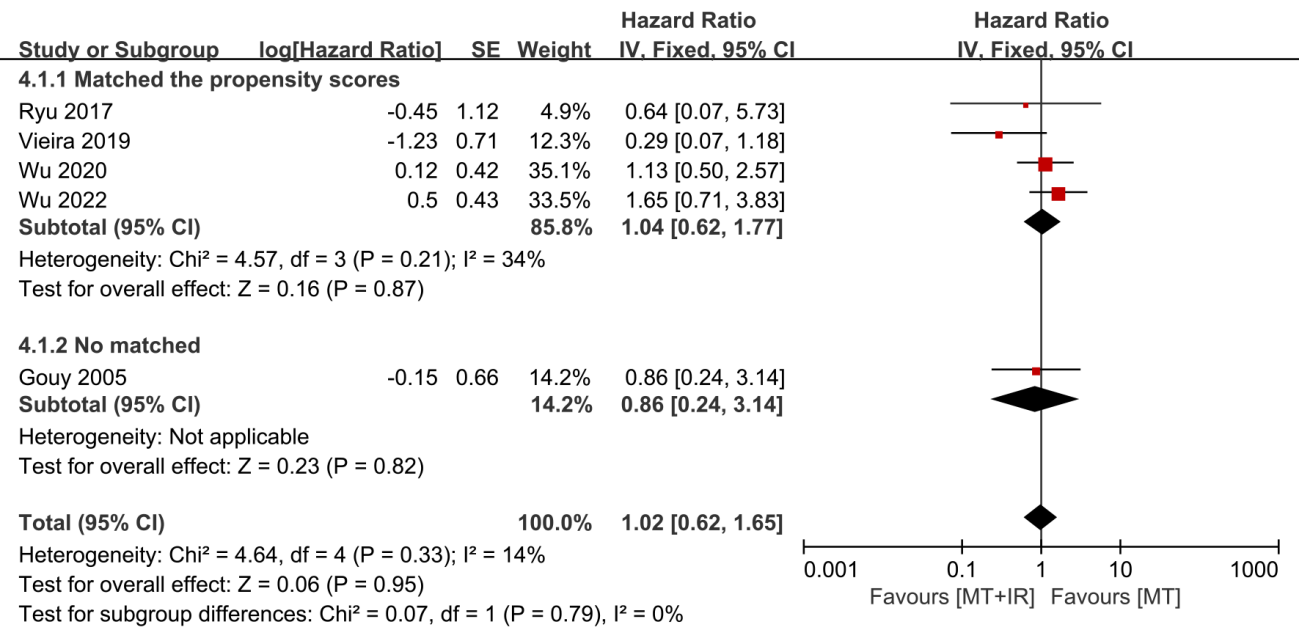


**Supplementary Figure 4.** Subgroup analysis of LRFS according to whether or not the propensity score was matched.


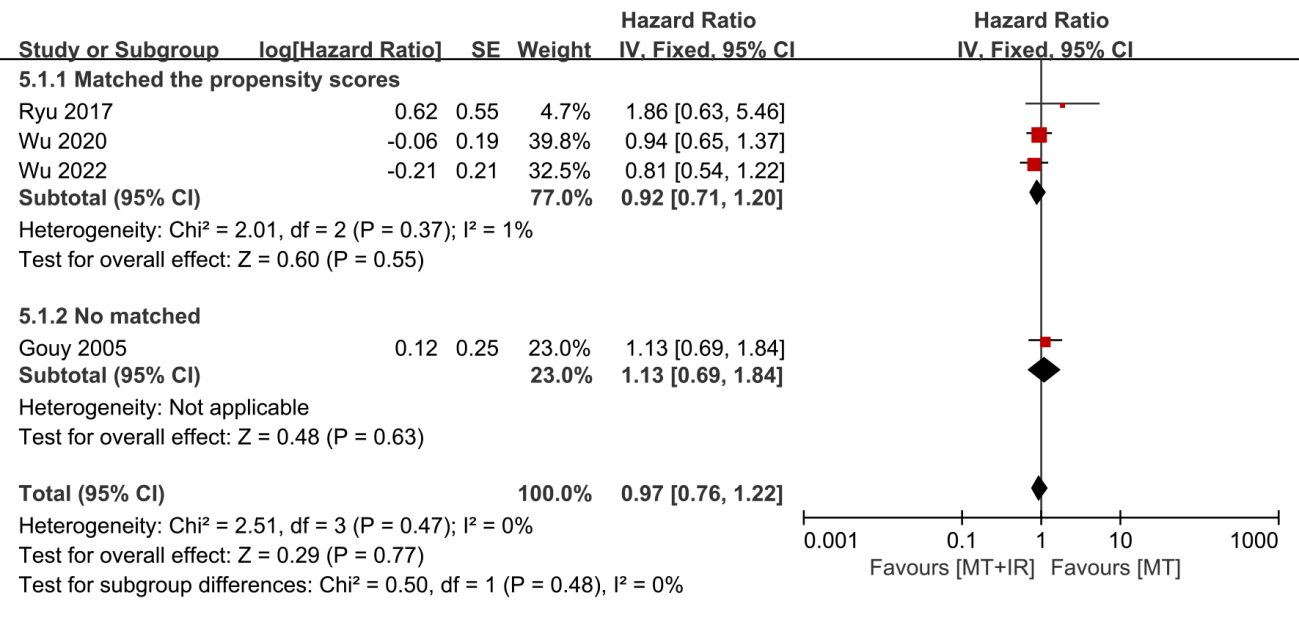


**Supplementary Figure 5.** Subgroup analysis of DMFS according to whether or not the propensity score was matched.
